# Role of cGAS/STING pathway in aging and sexual dimorphism in diabetic kidney disease

**DOI:** 10.1172/jci.insight.174126

**Published:** 2024-11-26

**Authors:** Sherif Khedr, Lashodya V. Dissanayake, Ammar J. Alsheikh, Adrian Zietara, Denisha R. Spires, Romica Kerketta, Angela J. Mathison, Raul Urrutia, Oleg Palygin, Alexander Staruschenko

**Affiliations:** 1 Department of Physiology, Faculty of Medicine, Ain Shams University, Cairo, Egypt.; 2 Department of Molecular Pharmacology and Physiology, University of South Florida, Tampa, Florida, USA.; 3 Department of Physiology, Medical College of Wisconsin, Milwaukee, Wisconsin, USA.; 4 Department of Physiology, Medical College of Georgia, Augusta University, Augusta, Georgia, USA.; 5 Genomic Sciences and Precision Medicine Center, Medical College of Wisconsin, Milwaukee, Wisconsin, USA.; 6 Division of Nephrology, Department of Medicine, Medical University of South Carolina, Charleston, South Carolina, USA.; 7 Hypertension and Kidney Research Center, University of South Florida, Tampa, Florida, USA.; 8 James A. Haley Veterans’ Hospital, Tampa, Florida, USA.

**Keywords:** Nephrology, Chronic kidney disease, Diabetes

## Abstract

Diabetic kidney disease (DKD) is the leading cause of chronic renal pathology. Understanding the molecular underpinnings of DKD is critical to designing tailored therapeutic approaches. Here, we focused on sex differences and the contribution of aging toward the progression of DKD. To explore these questions, we utilized young (12 weeks old) and aged (approximately 50 weeks old) type 2 diabetic nephropathy (T2DN) rats. We revealed that the cyclic GMP-AMP synthase (cGAS)/stimulator of interferon genes (STING) pathway was upregulated in T2DN rats compared with nondiabetic Wistar rats and in type 2 diabetic human kidneys. The activation of the cGAS/STING signaling pathway exhibited distinct protein expression profiles between male and female T2DN rats, with these differences becoming more pronounced with aging. RNA-Seq analysis of the kidney cortex in both male and female T2DN rats, at both younger and older ages, revealed several key molecules, highlighting crucial genes within the cGAS/STING pathway. Thus, our study delved deep into understanding the intricate sexual differences in the development and progression of DKD and we propose the cGAS/STING pathway as an essential contributor to disease development.

## Abstract 2

The cGAS-STING pathway represents a new therapeutic target to ameliorate the progression of DKD to end-stage kidney disease.

## Graphical abstract

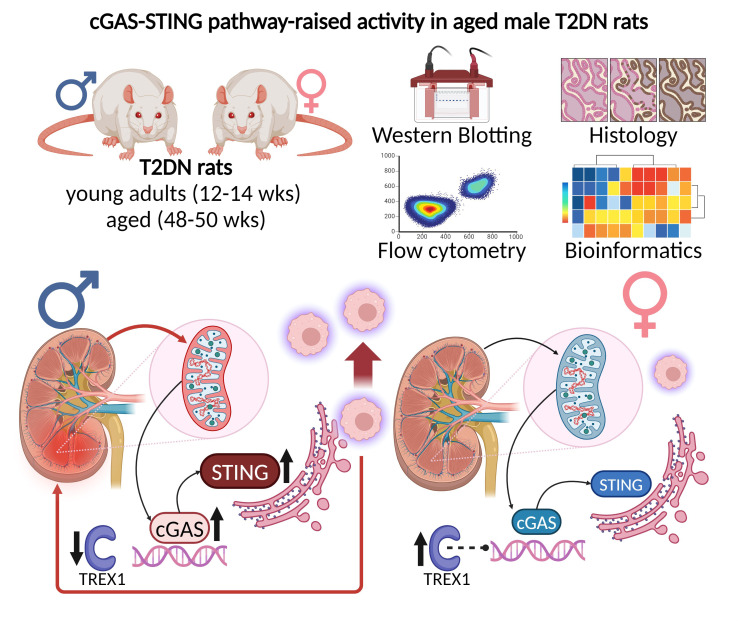

## Introduction

Innate immunity, as the body’s first line of defense, aims to limit the spread of infection and eradicate it altogether to preserve the integrity and functionality of all physiological systems. The immune system is heavily reliant on pattern recognition receptors that detect and identify pathogenic molecules, specifically those termed pathogen-associated molecular patterns, which then trigger specialized intracellular signaling cascades to coordinate innate immune mechanisms (1). This remarkable defense system works tirelessly to safeguard our health, underscoring the vital importance of immune health maintenance.

The innate immune nucleic acid sensors such as stimulator of interferon (IFN) genes (STING), retinoic acid–inducible gene I (RIG-I), melanoma differentiation-associated gene 5 (MDA5), or Toll-like receptors have evolved to recognize foreign pathogen-derived nucleic acids. This event provokes the translation of a plethora of host defense-related proteins like proinflammatory cytokines and chemokines. Meanwhile, the normal physiologic state is characterized by tight regulation of these mechanisms to avoid chronic inflammation pathology if persistently activated (2). Compartmentalization of self-DNA inside the nucleus and mitochondria, as well as the presence of efficient extranuclear DNA exonucleases, e.g., three prime repair exonuclease 1 (TREX1) capable of digesting aberrant cytosolic DNA, would blunt the activation of the immune system triggered by the accidental leakage of self-DNA (3, 4). However, under certain pathological conditions, these braking mechanisms are dysregulated, leading to a flareup of the inflammatory conditions, which are usually associated with massive organ destruction. Aicardi-Goutières syndrome (AGS) and systemic lupus erythematosus (SLE) are examples of diseases characterized by a severe progressive multiorgan inflammatory state commonly resulting in organ failure (5–7).

STING is an ER adaptor that has been discovered by high-throughput screening of a cDNA expression library (8). Sun et al. showed that STING could be galvanized after association with cyclic GMP-AMP (cGAMP) that is generated by cGAMP synthase (cGAS) following binding to cytosolic double-stranded DNA (9). The binding of cGAMP induces activation and conformational changes in STING, leading to its trafficking through the Golgi apparatus, with TANK-binding kinase 1 (TBK1) and activation of the transcription factor IFN regulatory factor 3 (IRF3), which in turn dimerizes and enters the nucleus to stimulate the transcription of type I IFN, as well as other proinflammatory cytokines, that fuels the innate immunity reaction (10). Since discovering the cGAS/STING pathway, a considerable number of studies have focused on its role in combating various microbial infections and carcinogenesis (11). However, its impact on other pathological conditions has not been adequately addressed. Despite this, its influence on diseases such as AGS, SLE, and myocardial infarction has been reported, and a dramatic prognostic improvement after either a pharmacologic or genetic interruption of this pathway has been demonstrated (12, 13).

Recent statistical data analysis points to the escalating prevalence of chronic kidney disease (CKD) among humans, affecting 1 out of 10 people (14), and it is well documented that diabetes is the leading cause of such a crippling health condition (15). While the global prevalence of type 2 diabetes is similar among both males and females, the results documenting the incidence of diabetic kidney disease (DKD) are controversial (16). Despite these alarming facts, there is a lack of efficient treatment to stop the CKD progression stemming from a poor understanding of the underlying mechanisms. A growing body of evidence shows that inflammation is a common sign of type 2 diabetes. It is characterized by an altered level of cytokines and chemokines and changes in the leukocyte number as well as their activation status (17, 18). However, the impact of the inflammatory cGAS/STING pathway on CKD has scarcely been studied. Chung et al. addressed the role of STING in the development of renal fibrosis and damage induced in CKD animal models and elegantly showed improvement in the outcome after pharmacological blocking or genetic knockout of STING in mice (19). Meanwhile, another group of researchers focused on STING as a key player in the acute kidney injury (AKI) animal model and showed the beneficial effects of impeding this pathway (20). Despite the divergence in the pathophysiologic mechanisms underlying CKD and AKI, it seems that the cGAS/STING pathway is an overlapping route.

Interestingly, recent studies show a strong relationship between STING activity and age-related cardiovascular and chronic lung disease (21, 22). Furthermore, activation of the STING pathway in diabetic *db/db* mice and mice with experimental Alport syndrome was recently reported (23). It was proposed that STING activation leads to podocyte injury and death. Another recent study confirmed that the mitochondrial DNA (mtDNA)/cGAS/STING pathway promotes podocyte injury and is a potential therapeutic target for DKD (24). The crucial role of STING in APOL1-associated podocytopathy was further proposed (25, 26). However, to our knowledge, the activity of the cGAS/STING pathway in aging and sexual dimorphism in DKD associated with type 2 diabetes has not been described previously. In our study, we explored the activation level of this inflammatory pathway using human kidney samples and an established rat model of type 2 diabetes with DKD, type 2 diabetic nephropathy (T2DN) rats (27, 28). Our data revealed a substantial increase in activity in the inflammatory cGAS/STING pathway associated with type 2 diabetes in the kidneys of T2DN rats. This activity increased with aging and was significantly higher in males compared with their age-matched females. The T2DN model exhibits significant differences in the progression of diabetes and kidney damage, with a notably protective phenotype observed in females. While both male and female T2DN rats develop hyperglycemia, insulin resistance, and glomerular damage, including substantial loss of nephrin and podocytes, females experience a milder form of these pathological changes, resulting in a slower disease progression, similar to what is seen in human conditions. This protective effect in females cannot be fully explained by hormonal differences alone, indicating the need for further investigation. Our findings suggest that the cGAS/STING pathway’s role in mediating innate immune responses is a crucial driver of DKD progression. Consequently, targeting the cGAS/STING pathway holds potential as a therapeutic strategy to slow the progression of DKD toward end-stage renal failure.

## Results

### Kidney-infiltrating leukocytes are the primary source of STING in human kidneys with type 2 diabetes and T2DN rats.

**Figure 1:**
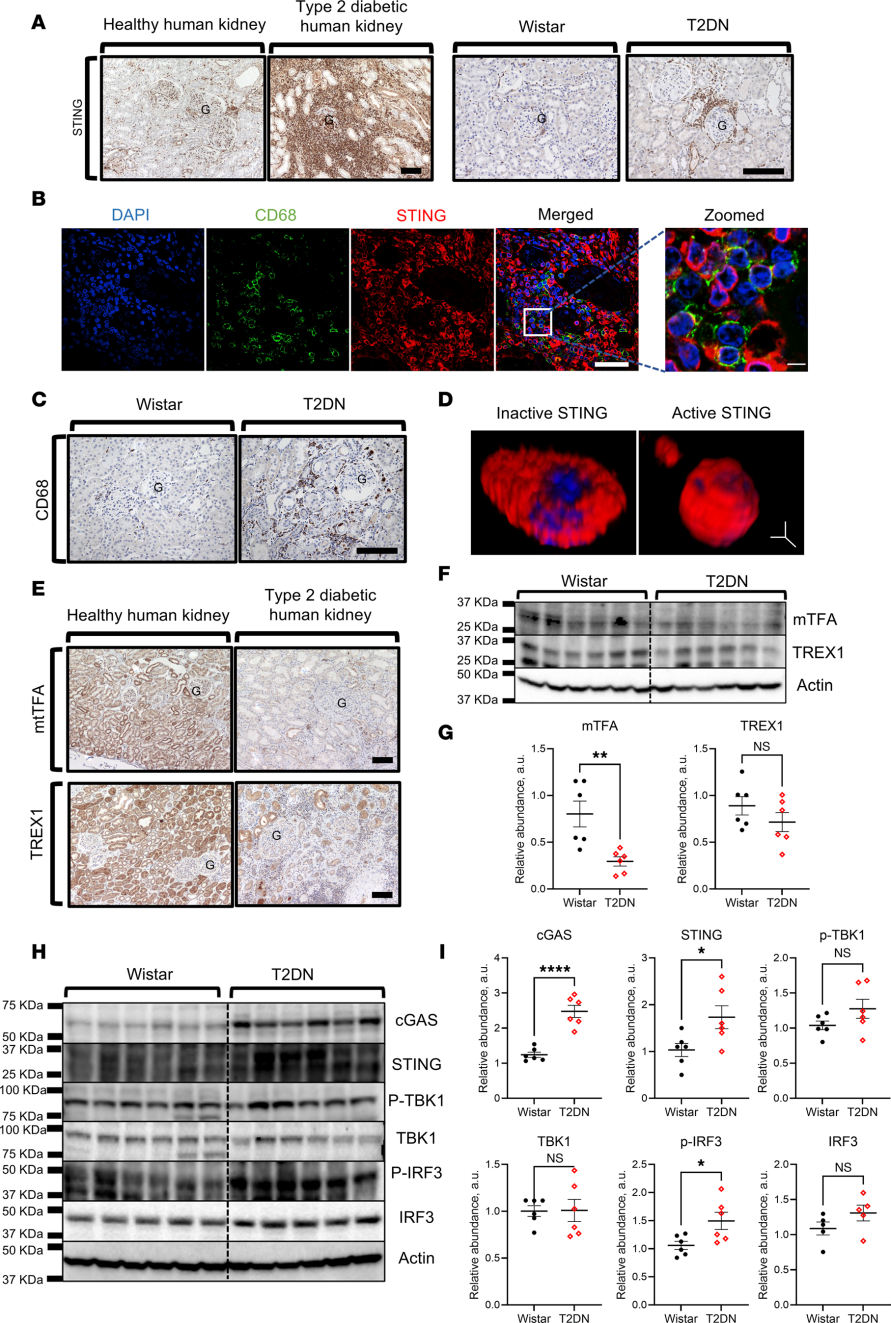
The expression pattern of cGAS/STING pathway molecules, TREX1 and mtTFA, in human and rat kidneys. (**A**) Representative images of immunohistochemical staining of kidneys for STING in healthy and type 2 diabetic humans (left), nondiabetic (Wistar) rats, and rats with type 2 diabetic neuropathy (T2DN) (right). G, glomerulus. Scale bars: 150 μm. (**B**) Examination of CD68 and STING by immunofluorescence microscopy in diabetic human kidney. Scale bars: 50 μm (left) and 5 μm (right). (**C**) Immunohistochemical detection of CD68 in kidneys of old male Wistar and T2DN rats. Scale bar: 150 μm. (**D**) Representative 3D image from a human kidney stained for inactive and active STING molecules. Scale bar: 2 μm. (**E**) Immunohistochemical staining for mtTFA and TREX1 in renal cortical sections from healthy and diabetic human kidneys. Scale bars: 150 μm. (**F**) Western blot analysis of mtTFA and TREX1 in the kidneys of Wistar and T2DN old male rats. *n* = 6 rats in each group. β-Actin was used as a loading control. (**G**) Summary graphs of relative abundance for Western blots shown above. (**H** and **I**) Western blot analysis of the cGAS/STING pathway molecules (cGAS, STING, p-TBK1, TBK1, p-IRF3, and IRF3) in the kidneys of Wistar and T2DN old male rats. *n* = 6 rats in each group. Data presented as mean ± SEM. Statistical analysis was performed using unpaired, 2-tailed Student’s *t* test. *P < 0.05, **P < 0.01, ****P < 0.0001. NS, nonsignificant; a.u., arbitrary units.

We first tested the expression and location of STING in the kidneys of type 2 diabetic and nondiabetic human patients using immunohistological staining (Figure 1A). Diabetic human kidneys showed a striking STING staining confined to non-renal infiltrating cells, which was almost absent in the healthy human kidneys. The substantial number of STING-loaded infiltrating cells obscured the renal histological architecture in many areas. A similar pattern was also seen in 50-week-old male T2DN rat kidneys. In contrast, the healthy human and control kidneys from old male Wistar rats did not express STING or show signs of cellular invasion (Figure 1A). Therefore, we show here the strikingly elevated expression of STING in type 2 diabetic kidneys in both humans and rats.

A significant proportion of cells expressing STING in the kidneys of diabetic patients and T2DN rats were positive for the macrophage marker CD68 (Figure 1, B and C). Interestingly, a perinuclear distribution of the STING molecule was also observed using immunofluorescence in both human diabetic and T2DN rat kidneys (Figure 1, B and D, Figure 5C, and Supplemental Figure 1A; supplemental material available online with this article; https://doi.org/10.1172/jci.insight.174126DS1). This distinctive organization of STING within the intracellular region reflects its activation status, adding to the complexity of this molecule’s involvement in DKD.

### Altered expression of mitochondrial transcription factor A, TREX1, and activity of the cGAS/STING pathway in diabetic kidneys.

Both mitochondrial transcription factor A (mtTFA) and TREX1 have been implicated in modulating the cGAS/STING pathway. Thus, we explored the expression level of mtTFA and TREX1 in human and rat kidneys using immunohistochemistry and Western blotting. Consistent with the CKD animal models described in the literature (19), we observed a significant reduction in mtTFA in the diabetic human kidney and T2DN rats compared with their controls (Figure 1, E–G). TREX1 expression was also decreased in the human diabetic kidney compared with the healthy one (Figure 1E). However, only a trend in reduction of TREX1 expression, which was not significant, was observed in T2DN rats (Figure 1, F and G). Western blot analysis of the kidneys isolated from 50-week-old male T2DN and Wistar rats revealed a significant increase in the expression and activation of the molecules involved in the cGAS/STING pathway, i.e., cGAS, STING, and IRF-3 (and their phosphorylated forms; Figure 1, H and I).

### Influence of sex and aging on kidney injury in T2DN rats.

**Figure 2:**
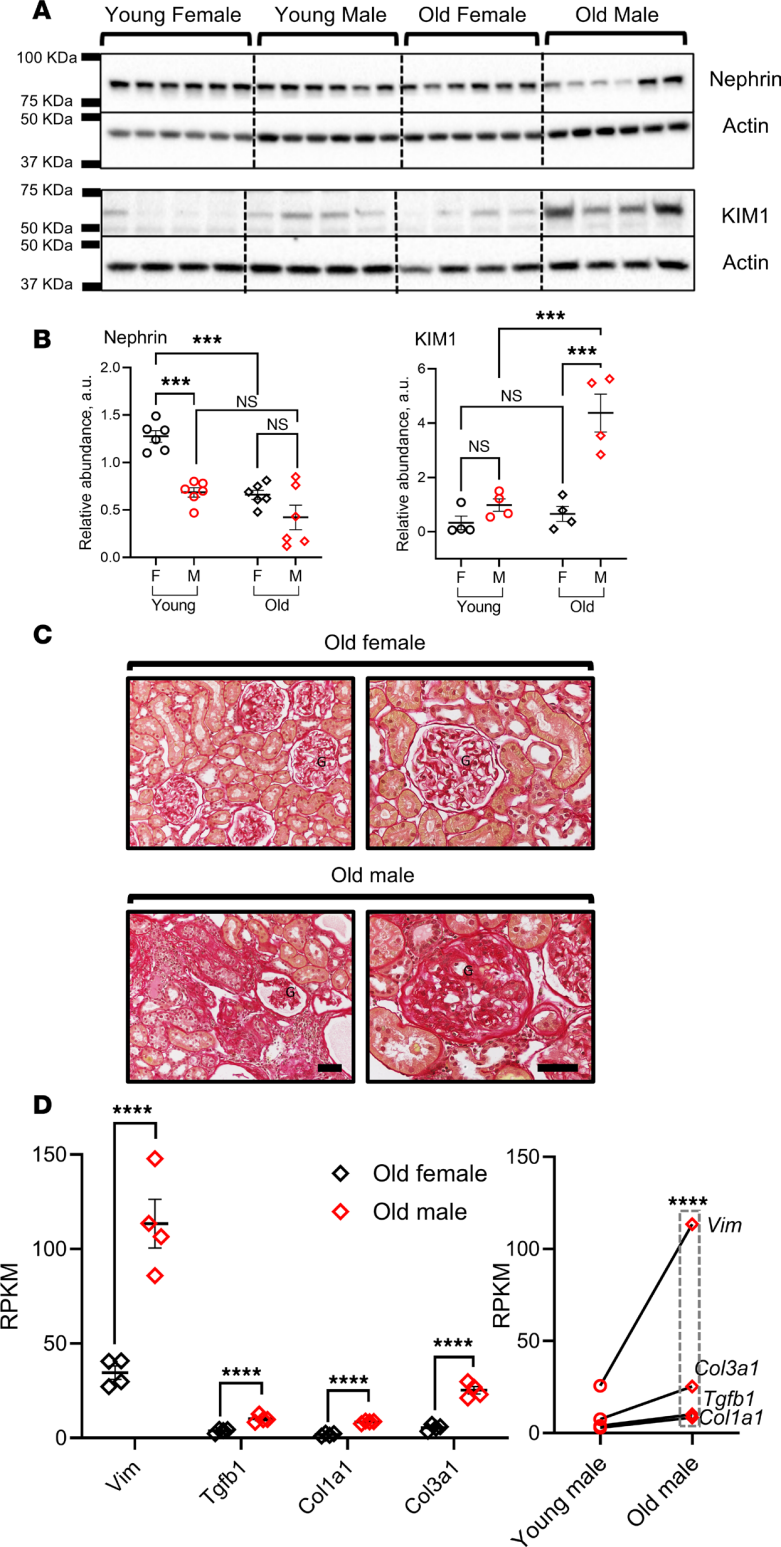
Differential levels of renal injury in T2DN rats of different sex and age groups. (**A**) Renal cortical nephrin and KIM1 expression tested using Western blotting for both sexes in young and old T2DN rats. *n* = 6 rats for nephrin and 4 rats for KIM1, respectively. (**B**) Summary graphs of relative abundance of nephrin and KIM1. Each dot represents 1 rat. Data shown as mean ± SEM. Statistical analysis was performed using 2-way ANOVA. ^***^P < 0.001. NS, nonsignificant; a.u., arbitrary units. (**C**) Representative images of picrosirius red staining of old female and male T2DN rat kidneys. G, glomerulus. Scale bars: 150 μm. (**D**) Expression level of fibrosis-associated genes (*Vim*, *Tgfb1*, *Col1a1*, and *Col3a1*) obtained from RNA-Seq data represented as RPKM. *n* = 4 rats in each group. Statistical analysis was performed using unpaired and paired 2-tailed Student’s *t* tests. ****P < 0.0001 indicates a statistically significant difference within the same genes.

In the following experiments, we explored the impact of sex and aging on renal injury and cGAS/STING pathway activity in T2DN rats. Our data underscored an increased liability for kidney injury among males versus females, even at an earlier stage of disease progression. We observed a trend in reduction in cortical nephrin associated with a significant increase in kidney injury molecule 1 (KIM1) in old male T2DN rats compared with their age-matched females (Figure 2, A and B). These results were supported by the renal histologic examination using picrosirius red staining, which showed pronounced interstitial fibrosis and glomerulosclerosis in old males compared with old females (Figure 2C). Moreover, a significant increase in the old male transcriptional profile of the fibrosis-associated genes (i.e., *Vim*, *Tgfb1*, *Col1a1*, and *Col3a1*) obtained from RNA-Seq data agreed with our histological analysis (Figure 2D). In contrast, there was no significant difference between the young males and young females or between young females and old females regarding the transcriptional pattern of the fibrosis-associated genes (Supplemental Figure 1B).

### Sexual dimorphism in mtTFA and TREX1 expression fuels the activity of the cGAS/STING pathway in T2DN rats.

**Figure 3:**
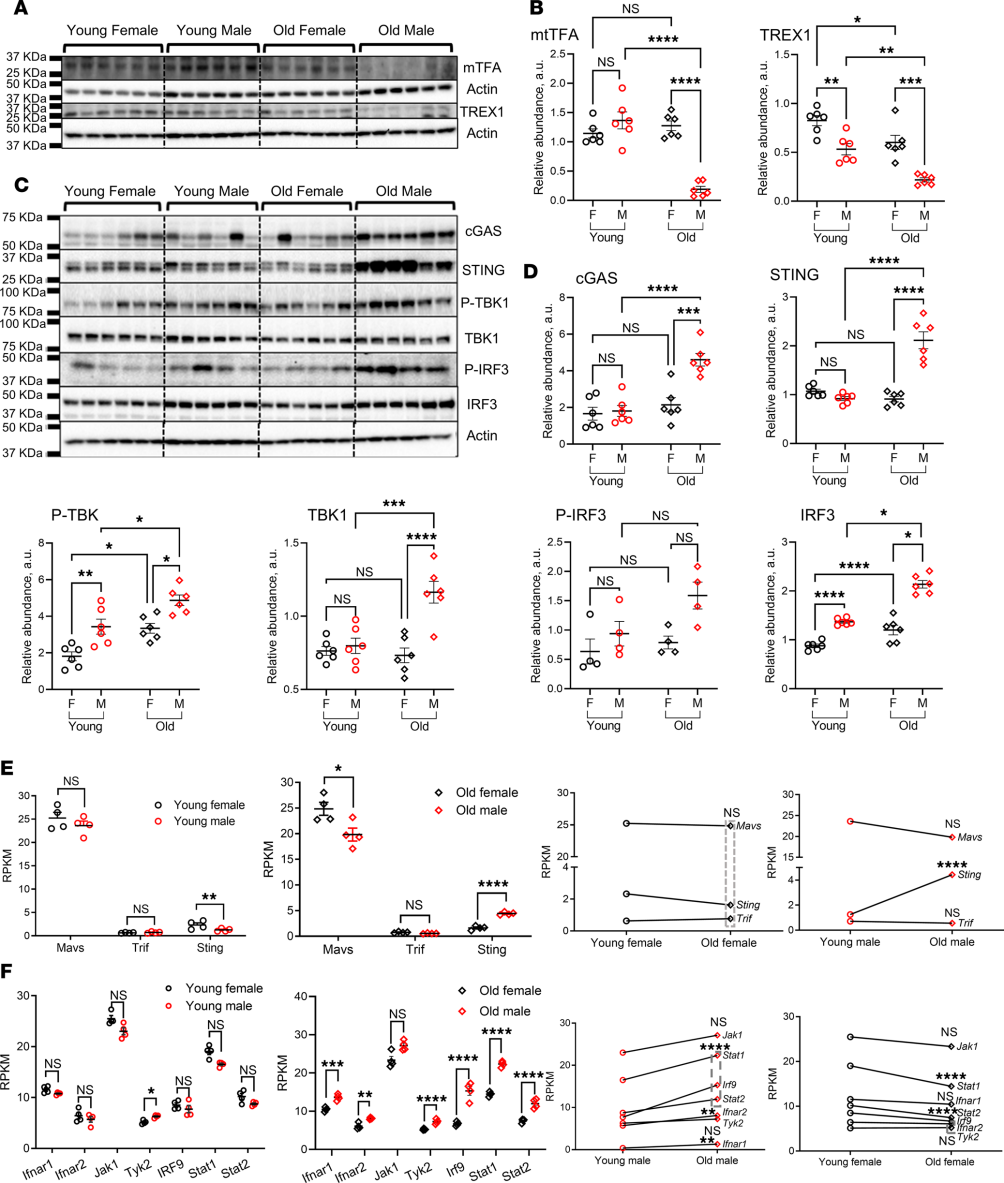
Expression analysis for TREX1, mtTFA, and cGAS/STING pathway molecules in T2DN rats of different sex and age groups. (**A**) Western blot analysis of mtTFA and TREX1 expression levels for both sexes in young and old T2DN rats; β-actin was used as a loading control. *n* = 6 rats in each group. (**B**) Summary graphs of relative abundance of mtTFA and TREX1 shown in **A**. Each dot represents 1 rat. (**C**) Expression level of the cGAS/STING pathway proteins (cGAS, STING, p-TBK1, TBK1, p-IRF3, and IRF3) in the kidneys of both sexes in young and old T2DN rats. Each lane represents 1 rat. *n* = 6, except for p-IRF3 in which *n* = 4. (**D**) Summary graphs of relative abundance of cGAS/STING pathway proteins after normalization to β-actin loading control. Data shown as mean ± SEM. Statistical analysis was performed using a 2-way ANOVA test. ^*^P < 0.05; ^**^P < 0.01; ^***^P < 0.001; ^****^P < 0.0001. NS, nonsignificant; a.u., arbitrary units. (**E**) IRF3 adaptor protein activator (*Mavs*, *Trif*, and *Sting*) levels obtained from RNA-Seq data represented as RPKM. Statistical analysis was performed using unpaired and paired 2-tailed Student’s *t* tests. *P < 0.05, **P < 0.1, ****P < 0.0001. (**F**) Type I IFN signaling pathway molecule (*Ifnar1*, *Ifnar2*, *Jak1*, *Tyk2*, *Irf9*, *Stat1*, and *Stat2*) levels obtained from RNA-Seq data represented as RPKM. *n* = 4 rats in each group. Statistical analysis was performed using unpaired and paired 2-tailed Student’s *t* tests. *P < 0.05, **P < 0.01, ***P < 0.001, ****P < 0.0001, indicates statistically significant difference within the same genes. NS, nonsignificant.

Intrigued by the expression levels of mtTFA and TREX1, we delved deeper into rat kidneys of varying age and sex groups using immunoblotting. Results showed no significant difference in the level of mtTFA in young males’ versus females’ kidneys. However, as time and disease progressed, we noted a substantial decrease in mtTFA expression levels in old male rats, making it significantly lower than the levels in old female rats and young male rats. Meanwhile, mtTFA was relatively stable without much fluctuation between the different female groups despite aging (Figure 3, A and B). The expression of TREX1, in general, was significantly lower in males versus their age-matched female group. Interestingly, both sexes showcased a significant decrease in TREX1 levels as they grew older (Figure 3, A and B).

Since mtTFA and TREX1 act as safety valves against stimulation of innate immunity through DNA sensors caused by self-leakage of mtDNA (29), it was plausible to investigate changes in the activity of the cGAS/STING pathway. Our results divulged a significant increase in the expression and the activity status of all molecules involved in the cGAS/STING pathway (i.e., cGAS, STING, total and p-TBK1, total and p-IRF3) in the old male group compared with the old female and young male groups. Except for the p-TBK1 and total IRF3, the females maintained their expression and activity levels for the different components of the cGAS/STING pathway over time, which were not affected by the aging process (Figure 3, C and D).

Previous studies showed that adaptor proteins MAVS, STING, and TRIF could activate the downstream protein kinase TBK1, which phosphorylates the transcription factor IRF3; thus, it controls type I IFN production (30). To demonstrate the impact of various adaptor proteins, we examined all 3 adaptors’ differential gene transcription. The data showed no difference in *Mavs* and *Trif* transcription among the young female and male groups. However, *Sting* was significantly higher in young females than their age-matched males. Interestingly, aging changed the transcriptional pattern to a significant reduction in *Mavs* and a marked increase in *Sting* in old male kidneys compared with old female kidneys. The two sexes showed nonsignificant temporal changes in the adaptor proteins, except for *Sting*, which increased more than 3-fold in the old male group with aging (Figure 3E).

The ultimate target of cGAS/STING pathway activation is to induce type I IFN production, which in turn acts in a paracrine fashion to stimulate the induction of IFN-stimulated genes (31–33). To confirm type I IFN downstream signaling activation, we checked the expression of all molecules involved in type I IFN signaling. Except for *Tyk2*, all other molecules (i.e., *Ifnar1*, *Ifnar2*, *Jak1*, *Irf9*, *Stat1*, and *Stat2*) showed a comparable transcriptional profile among both sexes at a young age. However, a significant increase was detected in *Ifnar1*, *Ifnar2*, *Tyk2*, *Irf9*, *Stat1*, and *Stat2* in the old male group compared with the old female group. The males showed significant increases in *Ifnar1*, *Ifnar2*, *Irf9*, *Stat1*, and *Stat2* with aging. In contrast, aging reduced *Stat1* and *Stat2* transcription in females, while not affecting the other molecules (Figure 3F).

### Old T2DN males exhibit renal inflammatory status.

**Figure 4:**
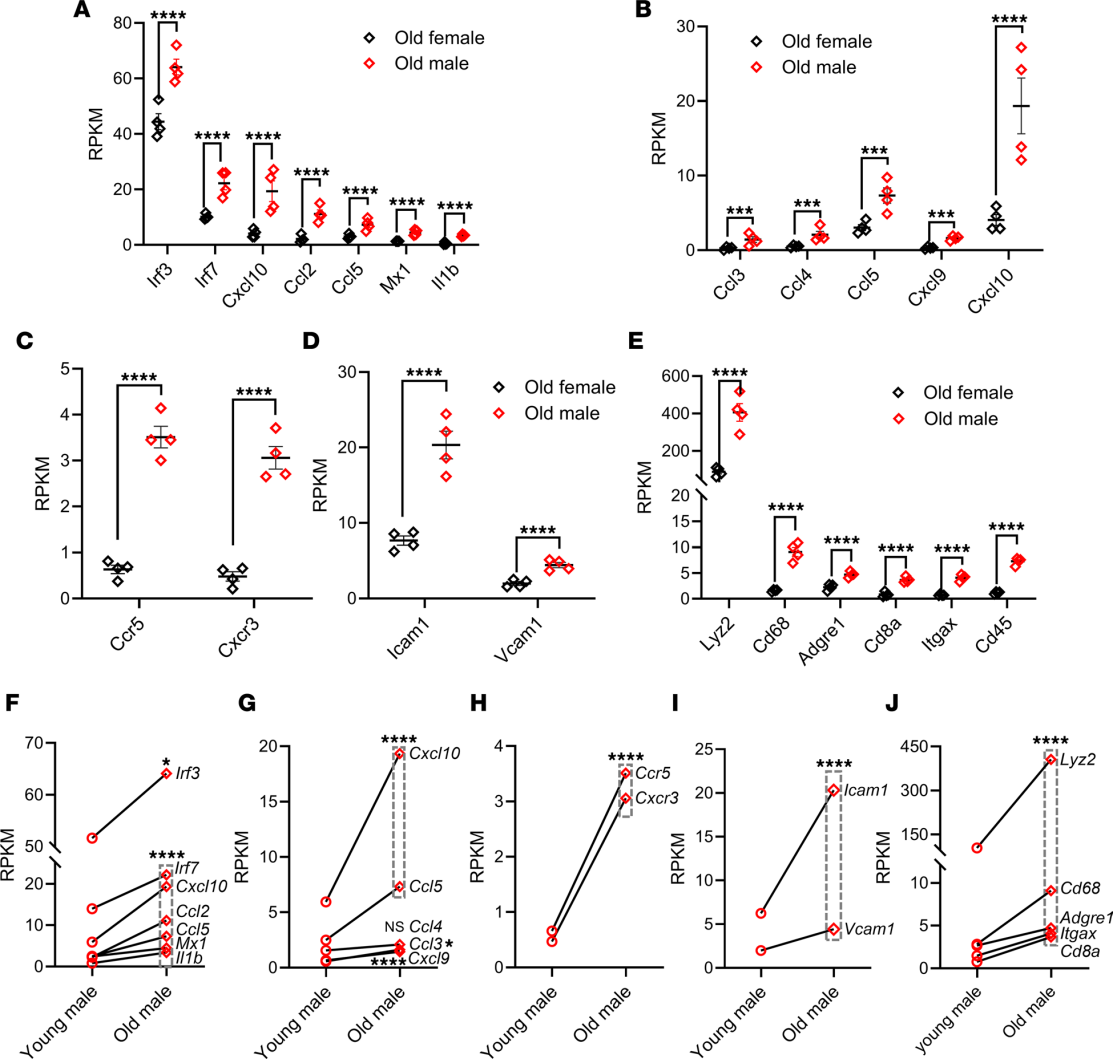
Differential inflammatory profile in T2DN rats of different sex and age groups. (**A** and **F**) Proinflammatory gene expression level. (**B** and **G**) Chemokine gene expression level. (**C** and **H**) Chemokine receptor gene expression level. (**D** and **I**) Adhesion molecule gene expression level. (**E** and **J**) Inflammatory cell marker gene expression level. Expression level obtained from RNA-Seq data represented as RPKM. Data are compared between old female and male T2DN rats (**A**–**E**) and young and old male rats (**F**–**J**). *n* = 4 rats in each group. Statistical analysis was performed using unpaired and paired 2-tailed Student’s *t* tests. *P < 0.05; ****P < 0.0001, indicates statistically significant difference within the same genes. NS, nonsignificant.

DKD has been established as an inflammatory disease (34, 35). In consensus with this fact, we observed a significant increase in the expression levels of proinflammatory genes, e.g., *Irf3*, *Irf7*, *Ccl5*, *Mx1*, *Il1b*, *Cxcl9*, and *Cxcl10*, in renal tissue of the old male group compared with the old female group (Figure 4A). In addition, several chemokines (i.e., *Ccl3*, *Ccl4*, *Ccl5*, *Cxcl9*, and *Cxcl10*) (Figure 4B) that are under the control of type I IFN, as well as their corresponding receptors (i.e., *Ccr5* and *Cxcr3*) (Figure 4C) (3, 31), showed a significant increase also in the old male group. As a logical consequence of this orchestrated sterile inflammatory status, the gene expression of adhesion molecules, i.e., *Icam1* and *Vcam1* (Figure 4D), as well as inflammatory cell markers (i.e., *Lyz2*, *Cd68*, *Adgre1*, *Cd8a*, *Itgax*, and *Cd45*) (Figure 4E), were significantly elevated in the old males versus old females. The aforementioned inflammatory signs increased significantly over time from the young to the old male group (Figure 4, F–J). In contrast, the females did not show any significant temporal changes; moreover, no sex difference could be detected between young age groups (Supplemental Figure 1, C–F).

### Effects of differential cGAS/STING activity in male versus female T2DN rats on renal immune cell infiltration.

**Figure 5:**
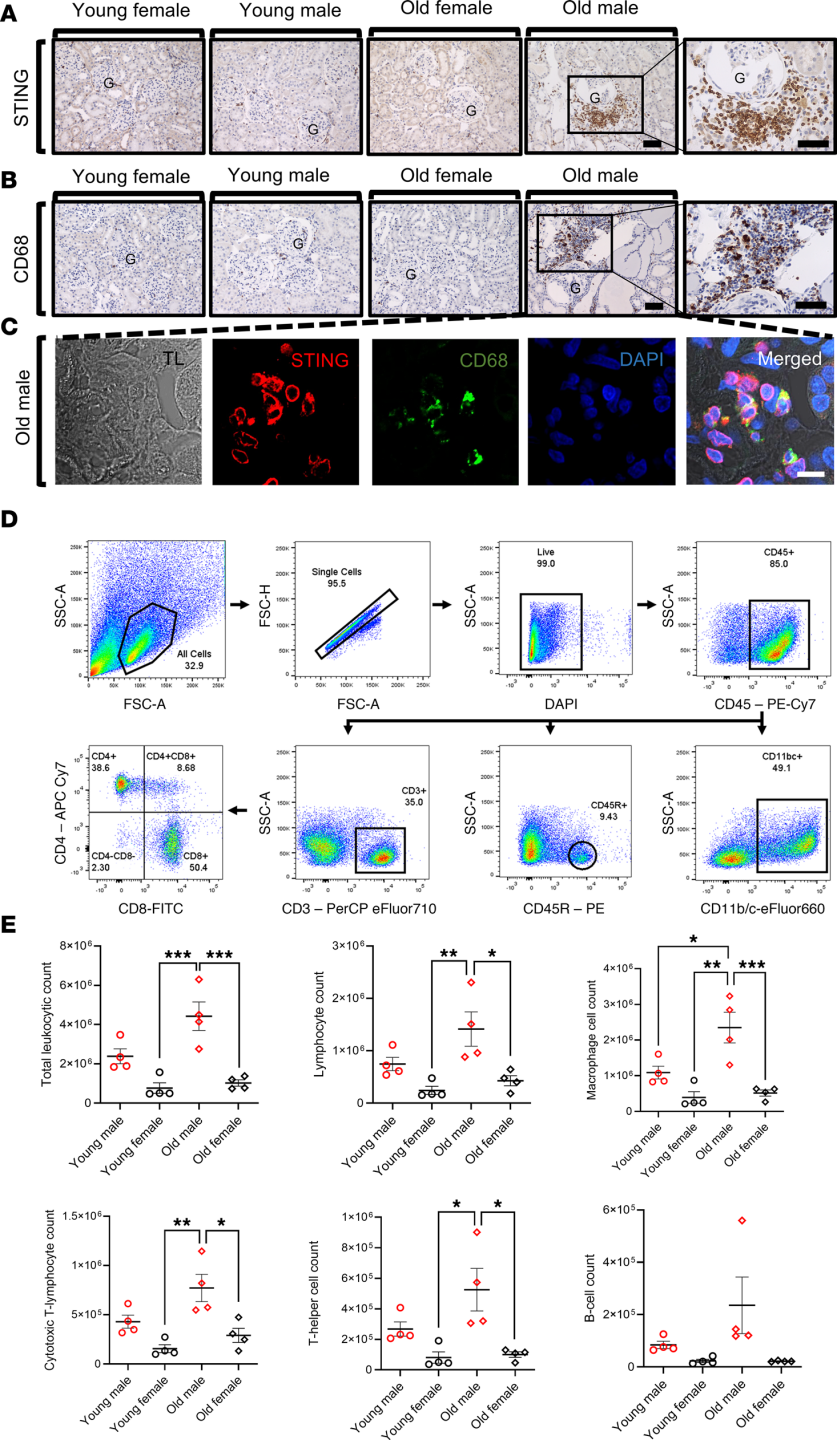
Flow cytometric analysis of the leukocytic renal infiltration in different groups. Representative images of immunohistochemical staining of STING (**A**) and CD68 (**B**) in T2DN rats of different sexes and ages. Scale bars: 150 μm. G, glomerulus. (**C**) Examination of CD68 and STING by immunofluorescence microscopy in the kidneys of old male T2DN rats. Scale bar: 10 μm. (**D**) Flow cytometric gating strategy for identification of leukocytes and their subsets from the kidney. (a) Two-parameter dot plots of forward versus side scatter was used to identify cells from debris. (b) The correlation between forward scatter area (FSC-A) and forward scatter height (FSC-H) identified single cells from doublets. (c) Live cells were gated as DAPI-negative. (d) From that, CD45^+^ total leukocytes were gated. Of the CD45^+^ cells, subpopulations were gated. (e) CD11b/c^+^ myeloid cells. (f) CD45R^+^ B lymphocytes. (g) CD3^+^ T lymphocytes. (h) Subpopulations of the CD3^+^ T lymphocytes of CD3^+^CD4^+^ T helper cells and CD3^+^CD8^+^ cytotoxic T cells. (**E**) Summary graphs of leukocytes and their subsets isolated from the kidney tissue of T2DN rats. Data are shown as mean ± SEM. Statistical analysis was performed using a 2-way ANOVA test. *n* = 4 rats in each group. *P < 0.05; **P < 0.01; ***P < 0.001.

Immunohistochemical analysis for STING in the renal tissue was done for all groups. The old male group showed evident STING-positive infiltrating cells invading the kidneys compared with minimal staining observed in the other 3 groups (Figure 5A). The intracellular perinuclear distribution of the STING molecule reflected its activation status (Figure 5C). Next, to ensure the leukocytic nature of these STING-positive infiltrating cells, immunohistochemical staining for CD68 was conducted, and there was a marked increase in macrophage count infiltrating the old male T2DN kidney (Figure 5, B and C).

Next, we explored the differential immune cell infiltration in the kidneys of all groups by a flow cytometry–based approach. A sample of the gating strategy used to identify leukocytes and their subpopulations is shown in Figure 5D. Results showed a significantly greater number of infiltrating leukocytes (CD45^+^) in the old male kidneys compared with their age-matched females. Moreover, the leukocytic count tended to increase in old males versus young males, while it was almost the same in females of different ages. Approximately 50% of the renal leukocytic population was monocytes/macrophages (CD11b/c^+^), with fewer numbers of CD3^+^ T cells, both CD3^+^CD4^+^ T helper and CD3^+^CD8^+^ cytotoxic T cells, and CD45R^+^ B cells (Figure 5E).

### RNA-Seq analysis of cGAS/STING pathway in T2DN rats.

**Figure 6:**
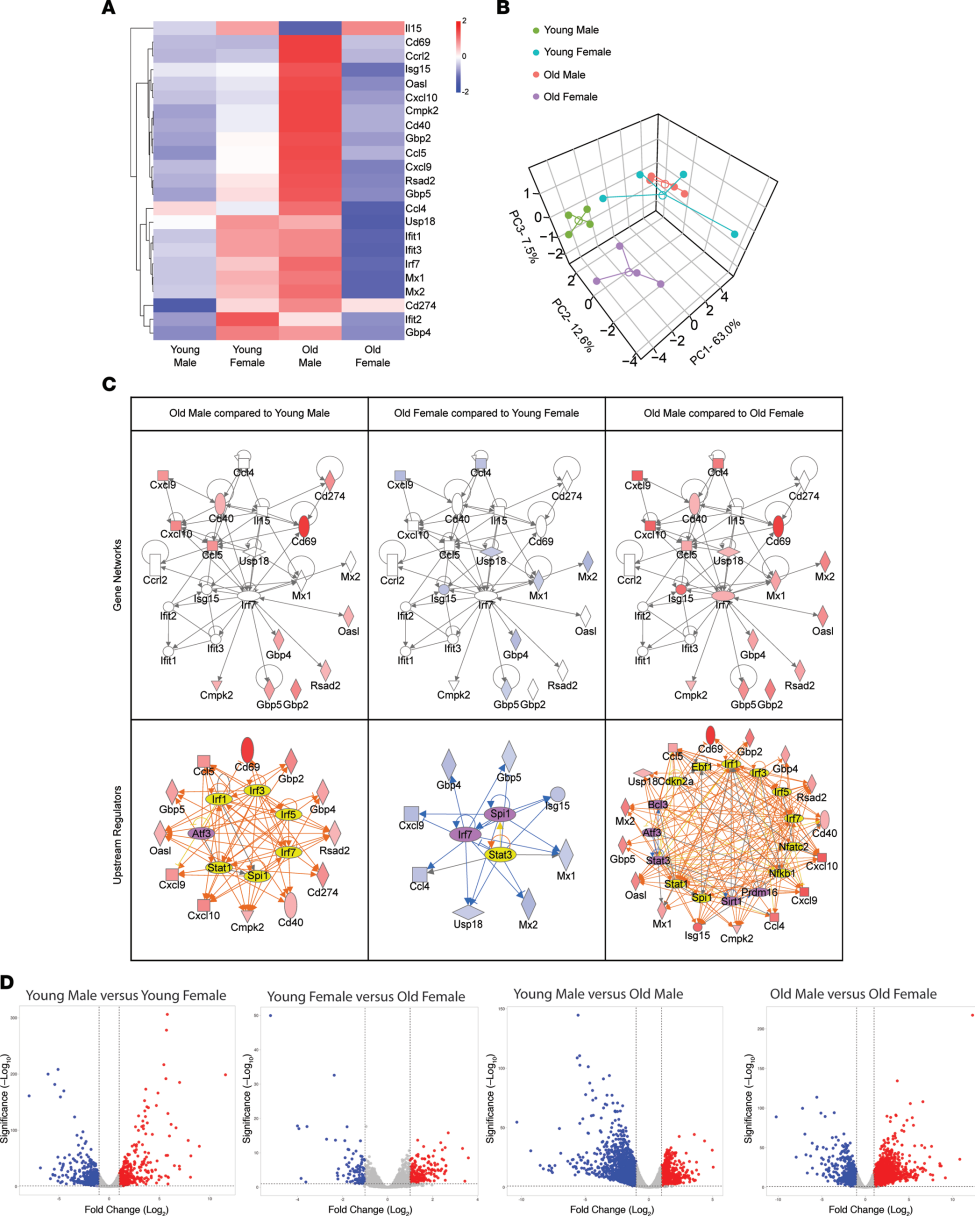
RNA-Seq analysis of cGAS/STING-specific genes. (**A**) RPKM expression level of genes normalized to the *z* scale. (**B**) PCA plot with centroids (open circles) and different animal groups (filled circles) using RPKM expression values of genes. (**C**) Gene networks and upstream regulatory analysis of cGAS/STING-specific genes for different animal groups. Gene networks: significant gene fold changes (|fold change| ≥ 2 and FDR < 0.05) with respect to controls are represented in red (upregulation) and blue (downregulation) nodes, while the nonsignificant genes have white nodes. Upstream regulatory analysis: color scheme of nodes is the same as in gene networks. Yellow and purple nodes represent predicted activated or inhibited transcription factors, respectively. Orange lines indicate predicted activation, blue lines indicate predicted inhibition, yellow lines indicate that the predicted relationship is inconsistent with gene expression, while gray lines indicate no predicted effect. (**D**) Volcano plots of statistically significant differentially expressed genes identified from the RNA-Seq data among different groups. *n* = 4 rats in each group.

**Figure 7:**
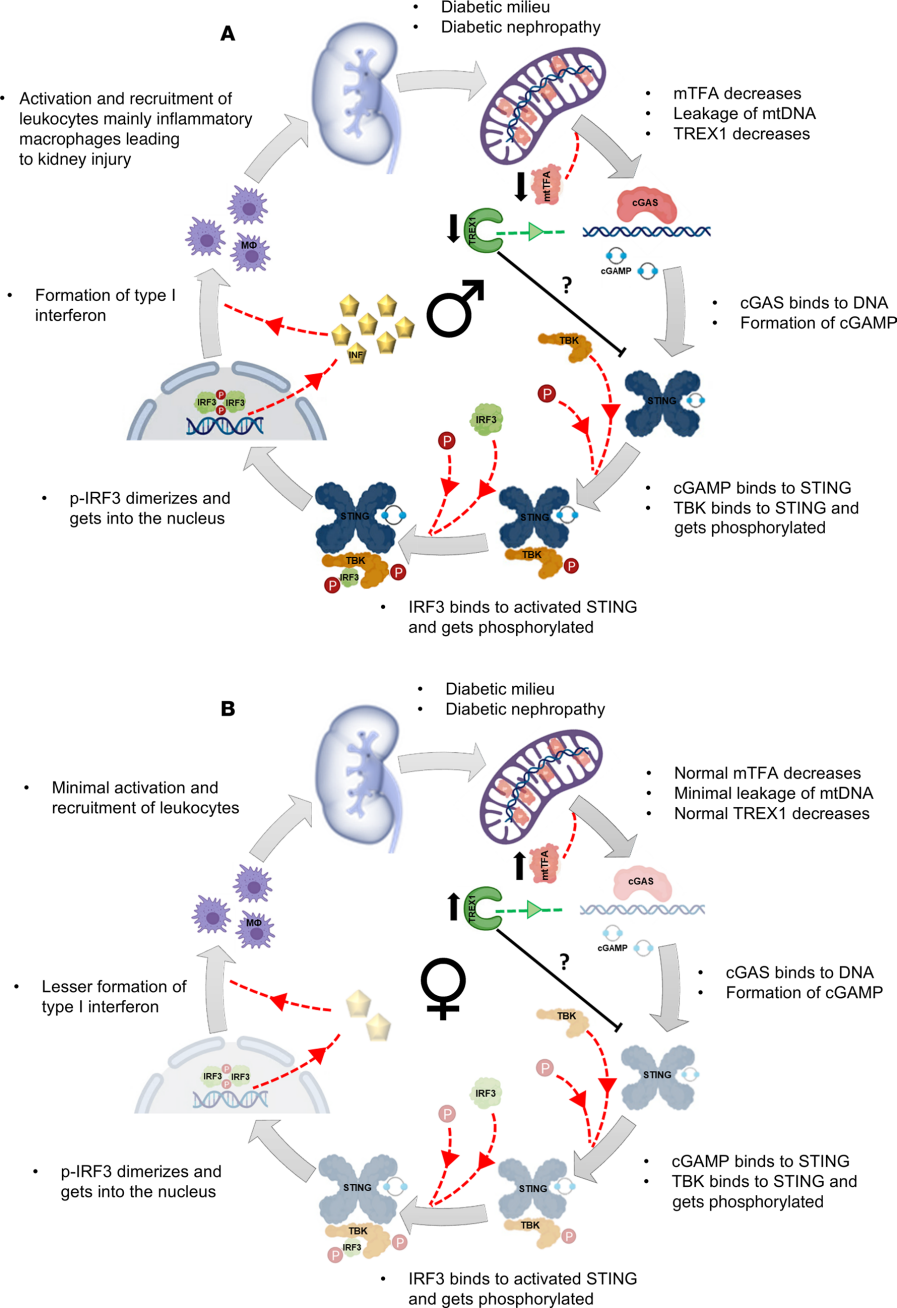
Summary of cGAS/STING activity. The results of this study are summarized schematically for male (**A**) and female (**B**) T2DN rats. The old male renal tissue showed clear signs of increased cGAS/STING pathway activity, with an elevation in IFN causing a significant inflammatory cell recruitment in comparison with their age-matched counterpart sex.

We further performed RNA-Seq analysis of genes participating in the cGAS/STING inflammatory pathway and plotted the gene expression values (reads per kilobase per million mapped reads, RPKM) for 23 genes that participate for the 4 different animal cohorts (Figure 6A). The analysis revealed that the old males displayed the highest level of gene expression than all other groups, indicating strong activation of genes in this pathway. Thus, concerning the sex, age differences, and diabetic disease progression, the older males had a higher expression of genes in this pathway than older females and younger males. We also performed principal component analysis (PCA) using RPKM expression values of the 4 different cohorts to assess the differences in gene expression patterns between the groups (Figure 6B). PCA of T2DN animals indicated that PC1 explains 63% of the data variance, while PC2 and PC3 explain 12.6% and 7.5%, respectively. We also found that the older males clustered the farthest from the older females and the younger males, with Euclidean distances of 9.25 and 7.51, respectively. The younger females appeared to cluster closer to the older males, with a Euclidean distance of approximately 5.13. Next, we performed a gene network and upstream regulatory analysis using the semantic-based algorithm Ingenuity Pathway Analysis (IPA; Figure 6C) (36). In agreement with gene expression data, we observed that genes in the cGAS/STING network were highly expressed in the older male cohort compared with younger males and older females (Figure 6C). Interestingly, some of these genes in the older female cohort were downregulated compared with younger females (Figure 6C), indicating that in females, there is an attenuation of the cGAS/STING pathway with age. A volcano plot was used to visualize the differential gene expression pattern between the 4 experimental groups (Figure 6D). The upstream regulatory analysis revealed predicted activation of transcription factors involved in IFN responses (*Irf1*, *Irf3*, *Irf5*, and *Irf7*) and other immune pathways (*Stat1*, *Spi1*, and *Nfatc2*) in older males compared with females and younger males. We also observed the downregulation of specific immune response transcription factors such as *Atf3*, *Bcl3*, and *Stat3*. Thus, data from this analysis indicate that the cGAS/STING pathway is highly active in older males compared with the other groups (Figure 7).

## Discussion

Chronic renal inflammatory status is known to cause metabolic, biochemical, and hemodynamic dysfunction that significantly contributes to the development and progression of DKD (30, 33, 35). It is also documented that the expression of inflammatory cytokines is higher in human CKD samples and animal models of kidney fibrosis, which further underscores the importance of keeping inflammatory status in check (19). Furthermore, recent studies revealed key contributions of the cGAS/STING pathway in glomerular pathology, including APOL1-associated podocytopathy (23–26). Genetic deletion of RIG-I or STING or treatment with a reverse transcriptase inhibitor ameliorates kidney fibroinflammation (37) and protects from cisplatin-induced kidney disease (38). Our study aimed to explore the activity of the inflammatory cGAS/STING pathway and the influence of sex and aging on its activity level in T2DN. Our results showed that the human kidney per se was unlikely a predominant source of STING under normal conditions. However, the uncontrolled hyperglycemic milieu imposed a sterile inflammatory status where STING became prominently expressed in non-renal infiltrating cells. These STING-loaded cells obscured the renal histological architecture in many areas. These cells’ leukocytic nature was confirmed, as they exhibited a pronounced expression of the macrophage maturation/activation marker CD68. Moreover, the perinuclear residence of the STING immunofluorescent staining in the infiltrating leukocytes reflected an activation status as STING translocates from the ER, the original resting location, to congregate in perinuclear, non-ER microsomal compartments after getting stimulated (39).

Our study utilized T2DN rats, the animal model for nonobese type 2 diabetes with DKD progression, mirroring clinical observations in human patients, including progressive glomerular damage (27). While the T2DN model captures key features of human disease, it is important to acknowledge the limitations inherent in translating these findings directly to clinical practice. Specifically, our ongoing and future studies targeting the role of sex hormones could provide important insights. The T2DN rats demonstrated renal inflammatory STING-loaded infiltrating cells, as seen in human kidneys with type 2 diabetic kidney disease. Our findings suggest that changes in mtTFA and TREX1 are a shared phenomenon in both renal tubules and infiltrating immune cells. As shown in Figure 1E, there was a significant reduction in mtTFA and TREX1 expression within both cell types. These findings align with previous research highlighting the critical role of TREX1 in mitigating inflammation. Specifically, *Trex1* knockout has been shown to exacerbate inflammatory responses, particularly in macrophages, through activation of the STING pathway (3). Furthermore, the accumulation of proinflammatory M1 macrophages has been implicated in the pathogenesis of DKD (40). Likewise, the healthy human and nondiabetic Wistar rat kidneys did not show signs of inflammation or STING-loaded cellular infiltration. The similarity between human and rat STING-associated pathology, as well as the human and rat STING structure (41), offers a promising avenue for further exploration of this pathway using the T2DN model.

The seminal work done by Ishikawa et al. showed that STING becomes galvanized following stimulation via sequence-nonspecific DNA (8). Recently, studies have documented the stimulatory influence of leaking mtDNA on the cGAS/STING pathway (42, 43). Meanwhile, Chung et al. showed that a reduction in mtTFA, a key regulator of gene expression in the mitochondria, in unilateral ureteral obstruction– and folic acid–induced CKD animal models leads to mtDNA leakage and subsequent cGAS/STING pathway activation (19). Therefore, we checked the expressional level of mtTFA in human renal tissue. The results showed a marked reduction in mtTFA in the diabetic kidney; hence, it is plausible that mtDNA leakage occurred. Thus, we investigated the level of TREX1, which acts as a safeguard against undesirable stimulation of the cGAS/STING pathway via digesting the aberrant cytosolic DNA (6). Interestingly, renal TREX1 expression was markedly affected in the diabetic human kidneys compared with the healthy ones. Although a similar pattern of effect was seen in T2DN rats, TREX1 only showed a reduced trend that did not reach a significant level. The mechanism underlying mtTFA and TREX1 reduction still needs to be clarified. We speculate that the depletion of renal mtTFA and TREX1 in diabetes paved the way for DNA to stimulate the cGAS/STING pathway. In agreement with our hypothesis, our results underscored a significant increase in cGAS/STING pathway activity in the kidneys of T2DN rats compared with Wistar rats.

Next, we deciphered the impact of sexual dimorphism and aging on renal diabetic injury in T2DN. Previous literature described the progressive nature of renal damage in uncontrolled diabetes (27), and our animal model followed the same route of escalating renal injury over time, which was revealed in the form of increased KIM1 in the kidneys and a reduction of cortical nephrin. Interestingly, the progression of renal damage showed a divergent sex-differential pattern (44). The old males were more susceptible to temporal renal injury than their age-matched females, i.e., more severe glomerulosclerosis, less cortical nephrin, elevated KIM1, and higher transcription of fibrosis-associated genes. Our previous study demonstrated a pronounced sexual dimorphism in T2DN rats, with females exhibiting milder disease phenotypes compared with males in terms of insulin resistance, lipid profile, renal function, and proteinuria (44). The observed sex-based disparity in the severity of disease might be attributable to the attenuated activation of the cGAS/STING pathway in female animals, potentially contributing to their milder clinical presentation.

One of the many reasons underlying the observed sex variation in kidney injury levels with aging might be the diversity in the inflammatory status level that could be attributed to the sexual dimorphism observed in the hyperglycemic milieu, insulin resistance, renal and glomerular injury, urinary nephrin shedding, and albumin handling (44). Therefore, checking for any sex-differential cGAS/STING inflammatory pathway activity was a strategic and logical extension of our work. As described above, the expressional levels of mtTFA and TREX1 were markedly lowered in the old male group compared with the young males and old females. The paucity of these two safety measures in old males fueled the activation of the cGAS/STING pathway, which was pronounced in the old male group compared with their age-matched females and young male group. It has been documented that the phosphorylation and, thus, activation of IRF3 by TBK1 occurs only with the assistance of an adaptor protein such as MAVS, STING, or TRIF (45). Data from the RNA-Seq study showed that the transcriptional copies of *Mavs* and *Trif* were not different between young males and females. However, *Sting* was significantly lower in the young male group compared with their age-matched female group. The transcriptional pattern of *Sting* changed with aging to be markedly elevated in the old male group compared with the old female group, which confirms our Western blot results.

The ultimate goal of cGAS/STING activation is to produce type I IFN for combating invading pathogens (46, 47). Our RNA-Seq data revealed a significant increase in the type I IFN downstream signaling molecules’ transcripts in the old male compared with the old female group. It is worth mentioning that these transcripts were of comparable values in both young male and female groups. However, both male and female groups showed a divergent transcriptional profile with aging; males exhibited a significant increase in *Ifnar1*, *Ifnar2*, *Irf9*, *Stat1*, and *Stat2*, and females showed a substantial reduction in *Stat1*, *Stat2*, and nonsignificant changes in all other transcripts. Interestingly, the significant elevation observed in the proinflammatory cytokines *Irf3*, *Irf7*, *Ccl5*, *Mx1*, *Ilb*, *Cxcl9*, and *Cxcl10* in the old males compared with the old females underscored the enhanced inflammatory status seen in this group. In agreement with this scenario, there was an associated increase in the chemokines *Ccl3*, *Ccl4*, *Ccl5*, *Cxcl9*, and *Cxcl10* that were under the control of type I IFN and their corresponding receptors *Ccr5* and *Ccr3*. Meanwhile, there was a concurrent increase in the expression of the adhesion molecules *Icam* and *Vcam*, with a subsequent increase in inflammatory cell markers in the old males. Again, the proinflammatory cytokines, chemokines and their corresponding receptors, and inflammatory markers displayed a temporal increase in males, while the females were remarkably stable over time.

Next, we checked the expression location of STING in the renal tissue of young and old males and females. Again, STING was nearly confined to non-renal infiltrating cells in the old male renal tissue; the leukocytic nature of these infiltrating cells was confirmed by the presence of CD68^+^ cells. Meanwhile, young and old females and young males did not show signs of cellular infiltration or STING staining. The perinuclear localization of STING in the old male group confirms the active status of this molecule.

Flow cytometric analysis for the total leukocytic infiltration revealed a significant difference between males and females. Kidneys from old male T2DN rats had higher leukocyte infiltration than kidneys from female rats. Macrophages, lymphocytes, cytotoxic T cells, and T helper cells were remarkably higher in the old male renal tissue than in the old females and young females. This infiltrating pattern mirrors the activation status observed in the cGAS/STING inflammatory pathway. A previous study demonstrated STING activation predominantly in the glomerulus of DKD in the *db/db* mouse model of DKD, but not in the tubules or due to any infiltrating cells (23). However, this study utilized relatively young mice, thus potentially limiting its ability to capture the full spectrum of disease progression. Other research using the same animal model has indicated a delayed onset of macrophage infiltration not before 6 months (48), aligning with the temporal profile observed in human diabetic nephropathy. By employing older rats with a more extended duration of diabetes, our study was able to observe a more advanced disease phenotype, closely resembling the patterns seen in human DKD.

Finally, the transcriptomic analysis for the different groups confirmed a pronounced higher activity of the cGAS/STING pathway in the old male rats compared with all other groups. This activity was associated with a marked elevation in the inflammatory profile, which explains the greater renal damage witnessed in the old male group.

Taken together, our data demonstrate that the cGAS/STING inflammatory signaling pathway contributes to the progression and magnitude of DKD, and its contribution depends on sex and is enhanced with aging. Therefore, pharmacologically inhibiting the cGAS/STING pathway and consecutively inflammation might be beneficial in treating DKD, especially in certain groups, such as aged males, where this pathway is highly elevated.

## Methods

### Sex as a biological variable

To investigate the impact of sex and age on the development of diabetic nephropathy, our study utilized both male and female rats of different age groups.

### Animals

T2DN rats of both sexes, either young adult (12–14 weeks) or old (48–50 weeks), were used in our experiments (27, 44). T2DN rats had been inbred for multiple generations at the Medical College of Wisconsin. Male Wistar rats (12 and 50 weeks, nondiabetic control) were purchased from Charles River Laboratories. Animals were fed a normal diet (5001, LabDiet, Purina) with water and food provided ad libitum on a 12-hour/12-hour light/dark cycle.

### Western blotting and antibodies

For Western blotting, kidney tissues were harvested after flushing with PBS via aortic cannulation (6 mL/min/kidney until blanched), and cortical sections were cut, weighed, and entirely dissolved in Laemmli buffer (1 M Tris-HCl pH 6.8, 10% SDS, 40% glycerol, 5% β-mercaptoethanol, 1% [w/v] bromophenol blue, dH_2_O) with protease inhibitors cocktail (Roche) and phosphatase inhibitor (phosStop, Roche) at 20 mg/mL with pulsed sonication for 5 to 10 seconds. Proteins were separated by SDS-PAGE and transferred onto a nitrocellulose membrane. Membranes were blocked with 5% (w/v) bovine serum albumin (BSA) in TBS containing 0.1% (v/v) Tween 20 for 1 hour before incubation overnight with the primary antibodies. The antibodies used were anti-mtTFA (Abcam, ab131607), anti-TREX1 (Abcam, ab83890), anti-cGAS (MyBioSource, MBS8291689), anti-STING (Cell Signaling Technology, 50494), anti–p-TBK1 (Cell Signaling Technology, 5483), anti-TBK1 (Cell Signaling Technology, 38066), anti–p-IRF3 (Cell Signaling Technology, 29047), anti-IRF3 (Cell Signaling Technology, 4302), anti-nephrin (Abcam, ab58968), anti-KIM1 (Cell Signaling Technology, 14971), anti-tubulin (ABclonal, ac030), and anti-actin (Santa Cruz Biotechnology, sc-1616). Primary antibodies were used at a dilution of 1:1,000. Secondary horse radish peroxidase–coupled anti-rabbit antibodies (Cell Signaling Technology, 7074) were used at a dilution of 1:10,000. Full immunoblots, including size markers, are shown in the supplemental material.

### Immunohistochemistry/immunofluorescence

For immunohistochemistry, formalin-fixed and paraffin-embedded kidney sections were deparaffinized and antigen retrieval was performed using H1(20) or H2(20) retrieval solution for 20 minutes at 100°C and then left to cool down to room temperature. For blocking, programmed steps ensued in the following order and durations: (a) wash buffer rinse (1 minute), (b) peroxidase block (15 minutes), (c) bond wash reagent (2 × 3 minutes), (d) avidin block (15 minutes), (e) bond wash reagent (2 × 3 minutes), (f) biotin block (15 minutes), (g) bond wash reagent (2 × 3 minutes), and (h) protein block (30 minutes; no rinse: blot/blow off reagent). (i) For detection, primary antibodies were incubated as follows: anti-STING (Cell Signaling Technology, 50494) was incubated at 1:50 overnight in H1(20), and secondary antibody was donkey anti-rabbit (1:500). Anti-CD68 (MilliporeSigma, MAB1435) was incubated at 1:100 for 30 minutes in H1(20), followed by secondary donkey anti-mouse (1:500). Anti-mtTFA (Abcam, ab131607) was incubated at 1:300 for 60 minutes in H1(20), and the secondary antibody was donkey anti-rabbit (1:500). Anti-TREX1 (Abcam, ab83890) was incubated at 1:50 for 90 minutes in H2(20), and the secondary antibody was donkey anti-rabbit (1:500). (j) After secondary antibodies, sections were washed with bond wash reagent (2 × 3 minutes), (k) incubated with biotinylated secondary antibody (anti-rabbit: Jackson ImmunoResearch, 711-066-152, 1:500; anti-mouse: Jackson ImmunoResearch, 715-066-151, 1:500) (30-minute incubation), (l) bond wash reagent (2 × 3 minutes), (m) DAKO tertiary (streptavidin-HRP; 1:300), (n) bond wash reagent (2 × 3 minutes), (o) DAB application (2 minutes), (p) distilled water rinse, and (q) counterstained with hematoxylin followed by bluing reagent, dehydrated, and then mounted with a coverslip.

Immunofluorescence was performed as previously described using fluorescein-conjugated secondary antibodies (49). Images were captured on a confocal Leica TCS SP5 inverted microscope using an HC Fluotar 25×/0.95 objective. Captured images were analyzed with ImageJ software (NIH).

### Histological staining

The rat kidneys were fixed in 10% paraformaldehyde for histological analysis, embedded in paraffin, and serially sectioned at 4 μm as previously reported (50). Slides were deparaffinized using xylene or a xylene substitute and hydrated through alcohols, and then rinsed with distilled water and placed in modified Meyer’s hematoxylin for 5 minutes. Following that, slides were rinsed in running tap water until nuclei were blue, and then placed in picrosirius red stain for 1 hour and rinsed with 2 changes of 0.5% acetic acid. Next, samples were dehydrated quickly through 3 changes of absolute alcohol and then cleared with 3 changes of xylene or xylene substitute. All the histological staining images were scanned with a Nikon Super CoolScan 9000 interfaced with NikonScan 4 software. Captured images were analyzed with ImageJ software.

### Kidney leukocyte isolation

Kidneys were flushed with heparinized PBS solution and extracted. Immune cell isolation, staining, and flow cytometry were performed to assess leukocyte infiltration in the kidney as we have previously described in detail (51, 52). After the kidney was flushed of blood and collected, the renal tissue was minced in a petri dish and passed through a 100-μm strainer (VWR, 89508-340) in RPMI-1640 medium containing collagenase type 4 (0.1%; Worthington, CLS-4) and DNase I (final conc. 10 μg/mL; Sigma-Aldrich, D5025), and the contents were then incubated in a temperature-controlled oven at 37°C for digestion for 30 minutes. The digested kidney tissue was then passed through a 70-μm strainer (BD Falcon, 352350) and centrifuged. Cells were resuspended in 10 mL of wash buffer (1× PBS with 2% FBS, 2 mM EDTA, and without Ca^2+^ and Mg^2+^) and then passed through a 40-μm strainer (VWR, 89508-342). Cells were then centrifuged and resuspended in 4 mL of 30% Percoll in RPMI-1640 and carefully layered over a 70% Percoll solution in PBS and then centrifuged to isolate mononuclear cells. The cell layer was then collected, resuspended in a wash buffer, and centrifuged. Finally, cells were resuspended in a known volume of wash buffer and then 10 μL aliquots were counted with a hemocytometer.

### Cell staining and flow cytometry

Approximately 1 × 10^6^ cells were aliquoted into 5 mL flow cytometry tubes (BD Falcon, 352052) and centrifuged. Cells were resuspended in 100 μL wash buffer and incubated with Fc Block (anti-CD32, BD Pharmingen, 550271) for 10 minutes. Cells were stained with a fluorochrome-bound antibody cocktail containing anti-CD45 PE-Cy7 (BioLegend, catalog 202214, clone OX-1; 0.062 μg), anti-CD3 PerCP eFluor710 (eBioscience, catalog 46-0030-82, clone G4.18; 0.062 μg), anti-CD8a FITC (BioLegend, catalog 201703, clone OX-8; 0.125 μg), anti-CD4 APC-Cy7 (BioLegend, catalog 201518, clone W3/25; 0.062 μg), anti-CD11b/c Alexa Fluor 660 (eBioscience, catalog 50-0110-82, clone OX-42; 0.062 μg), and anti-CD45R PE (BD Biosciences, catalog 554881, clone HIS24; 0.125 μg) in 100 μL of wash buffer (1× PBS with 2% FBS, 2 mM EDTA, and without Ca^2+^ and Mg^2+^) for 30 minutes. Excess antibodies were washed, and cells were resuspended in 300 μL of wash buffer. DAPI was added for the quantification of dead cells 10 minutes before running samples on a flow cytometer. Flow cytometry was then performed using either an LSR II flow cytometer or Fortessa X20 flow cytometer (both BD Biosciences) and gating and data analysis were performed by using FlowJo Software (FlowJo, LLC). The gating strategy used to identify different immune cell subsets is shown in Figure 5D.

### Bioinformatics

Reads were aligned to the rat reference transcriptome Rnor_6.0.98 (Ensemble), with at least 13.5 million mapped read pairs acquired per sample. Sequencing reads were processed through the GSPMC workflow, including MapRseq3 (53) and differential expression calculated by the edgeR Bioconductor package (54). Differentially expressed cGAS/STING genes were defined as genes with a false discovery rate (FDR) of less than 0.05 and an absolute fold change of 2 or greater between at least one treatment sample and the control sample. Gene network analysis was completed using IPA (36).

### Statistics

All data are expressed as mean ± SEM and were analyzed using GraphPad Prism 8.0, OriginPro (OriginLab Corp.), and SigmaPlot 14.5 (Systat Software, Inc. 2020). Significance was assessed by 2-way ANOVA followed by Tukey’s test or unpaired/paired Student’s *t* test, both 2-tailed. In all statistical comparisons, a *P* value of less than 0.05 was used to indicate a statistically significant difference.

### Study approval

#### Animal studies.

Animal use and welfare adhered to the NIH *Guide for the Care and Use of Laboratory Animals* (National Academies Press, 2011), following a protocol reviewed and approved by the IACUC at the Medical College of Wisconsin.

#### Human tissue.

Human tissue was obtained from deidentified patients from the Medical College of Wisconsin Tissue Bank as described previously (55).

### Data availability

RNA-Seq data are available in the NCBI Gene Expression Omnibus (GEO GSE243138) or via the following link: https://www.ncbi.nlm.nih.gov/geo/query/acc.cgi?acc=GSE243138 The data presented in this article, along with additional supporting data and supplemental information, are available online and upon request from the corresponding author. Raw data can be found in the Supporting Data Values file.

## Author contributions

SK conceptualized the study. SK, AJA, LVD, AZ, DRS, and OP carried out the investigation. SK, AJA, LVD, RK, AJM, and RU analyzed data. SK and AS wrote the original draft of the manuscript, which was reviewed, edited, and given final approval by SK, AJA, LVD, AZ, DRS, RK, AJM, RU, OP, and AS. AS provided resources and supervision. SK and AS are the guarantors of this work and, as such, had full access to all the data in the study and take responsibility for the integrity of the data and the accuracy of the data analysis.

## Supplementary Material

Supplemental data

Unedited blot and gel images

Supporting data values

## Version 1 11/26/2024

In-Press Preview

## Version 2 01/09/2025

Electronic publication
