# Supplementary material for: Role of cGAS/STING pathway in aging and sexual dimorphism in diabetic kidney disease
Source: JCI Insight. 2024 Nov 26;10(1):e174126. doi: 10.1172/jci.insight.174126 (PMC11721291; doi:10.1172/jci.insight.174126)
Supplement: Supplemental data [file jciinsight-10-174126-s127.pdf]

Supplemental Material

Role of cGAS-STING pathway in aging and sexual dimorphism in diabetic kidney disease

Sherif Khedr[1], Lashodya V. Dissanayake[2], Ammar J. Alsheikh[3], Adrian Zietara[2], Denisha R. Spires[4], Romica Kerketta[5], Angela Mathison[5], Raul Urrutia[5], Oleg Palygin[6], Alexander Staruschenko[2,7,8,*]

[1]Department of Physiology, Faculty of Medicine, Ain Shams University, Cairo, Egypt; [2]Department of Molecular Pharmacology and Physiology, University of South Florida, Tampa, FL 33602, USA; [3]AbbVie Inc, Cambridge, MA 02139, USA; [4]Department of Physiology, Medical College of Georgia, Augusta University, Augusta, GA 30912, USA; [5]Genomic Sciences and Precision Medicine Center, Medical College of Wisconsin, Milwaukee, WI 53226, USA; [6]Division of Nephrology, Department of Medicine, Medical University of South Carolina, Charleston, SC 29425, USA; [7]Hypertension and Kidney Research Center, University of South Florida, Tampa, FL 33602, USA; [8]James A. Haley Veterans Hospital, Tampa, FL 33612, USA

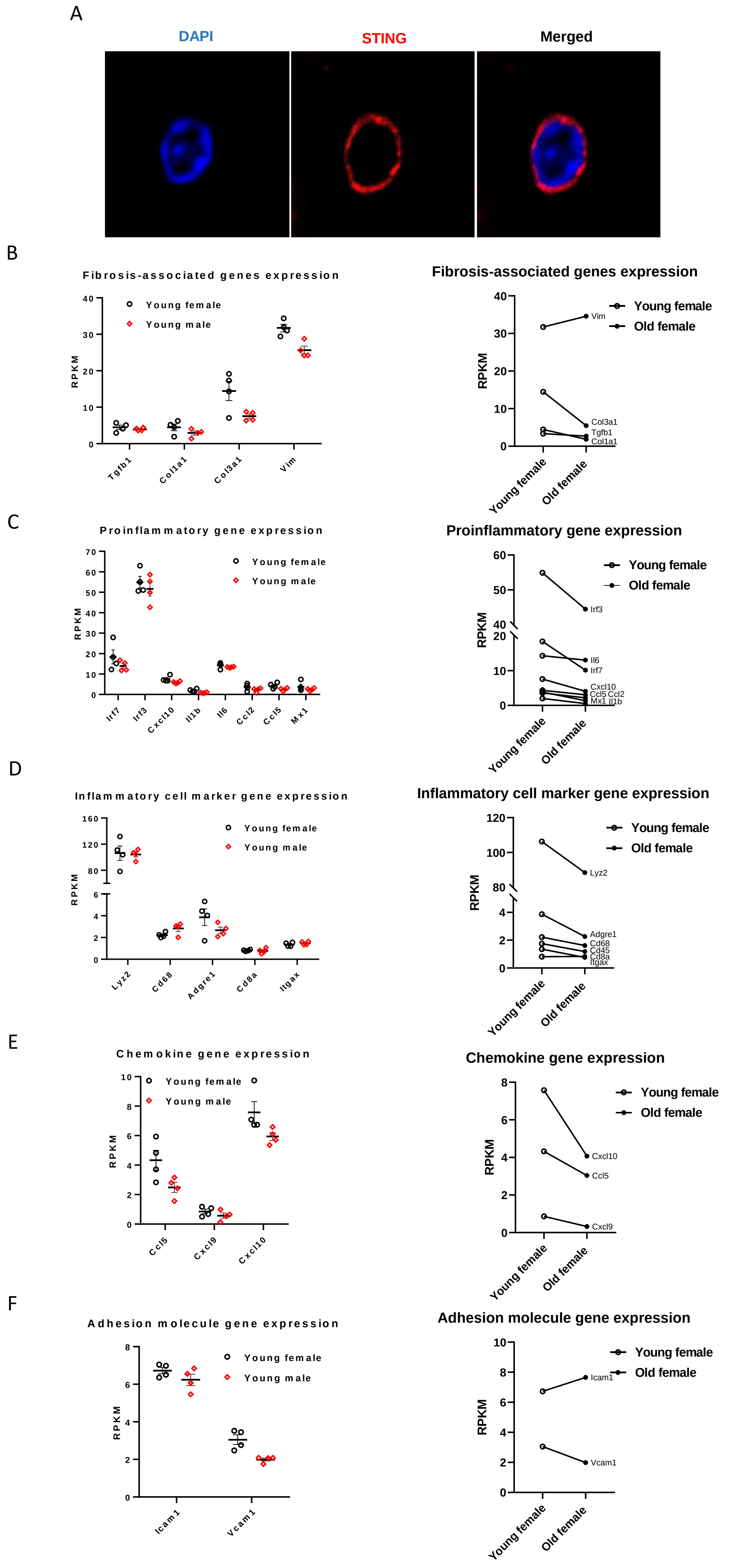

Supplementary Figure 1 | (A) 2D image showing perinuclear localization of the STING molecule. **(B)** The expression level of fibrosis-associated genes. **(C)** Pro-inflammatory gene expression level. **(D)** Inflammatory cell markers gene expression level. **(E)** Chemokine gene expression level. **(F)** Adhesion molecules gene expression level. Expression level obtained from RNA-Seq data represented in RPKM. NS, non-significant.
