# Supplementary material for: Role of cGAS/STING pathway in aging and sexual dimorphism in diabetic kidney disease
Source: JCI Insight. 2024 Nov 26;10(1):e174126. doi: 10.1172/jci.insight.174126 (PMC11721291; doi:10.1172/jci.insight.174126)
Supplement: Unedited blot and gel images [file jciinsight-10-174126-s128.pdf]

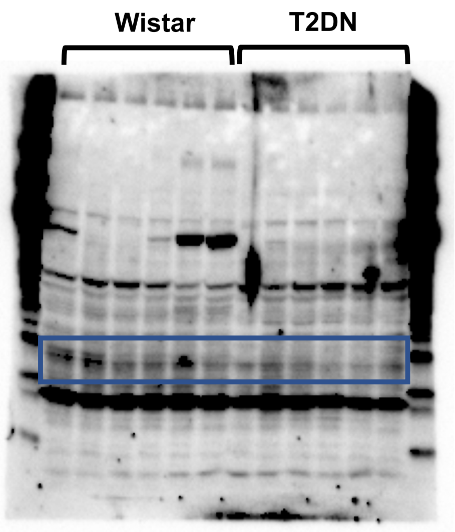

mTFA (ab131607)

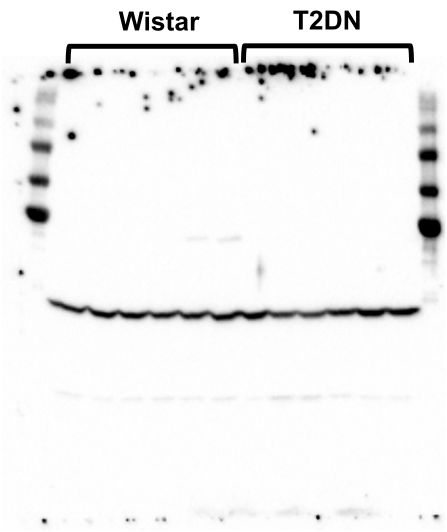

Actin (sc-1616)

Full unedited gel for Figure 1F

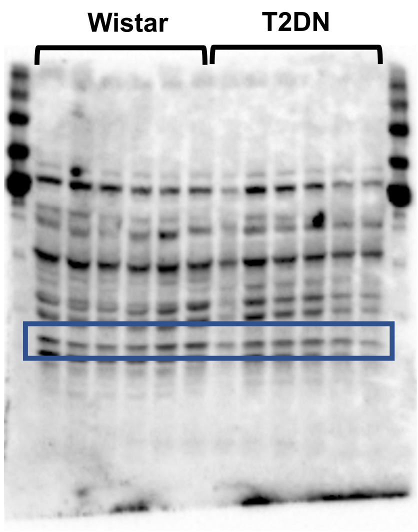

TREX1 (ab83890)

Full unedited gel for Figure 1F

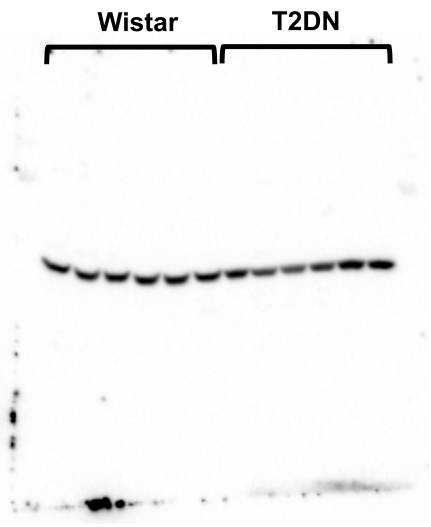

Actin (sc-1616)

Full unedited gel for Figure 1F

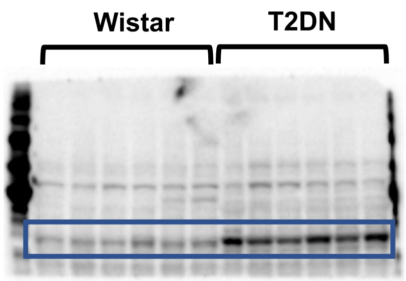

cGAS (MBS8291689)

Full unedited gel for Figure 1H

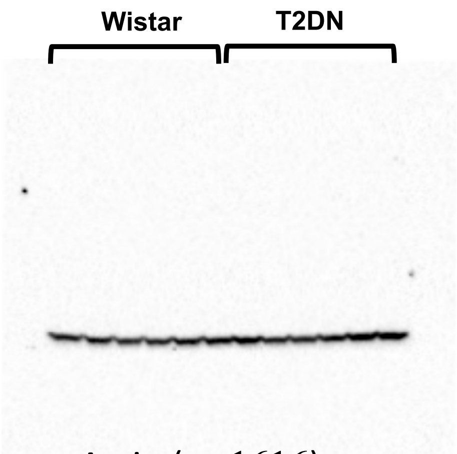

Actin (sc-1616)

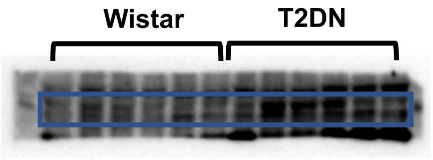

STING (CST50494)

Full unedited gel for Figure 1H

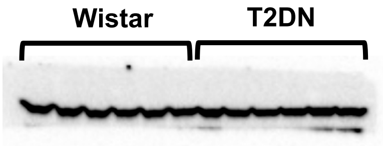

Actin (sc-1616)

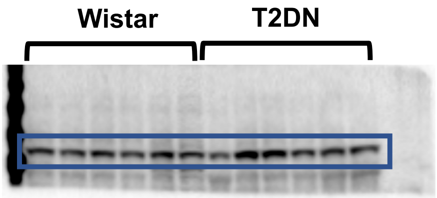

P-TBK1 (CST5483)

Full unedited gel for Figure 1H

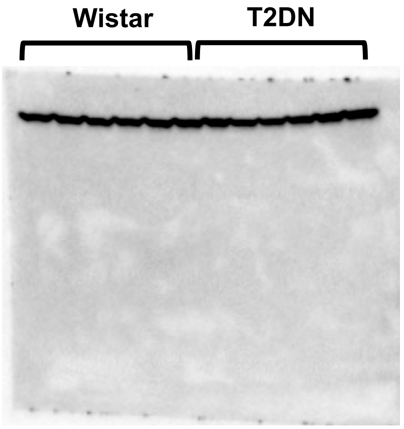

Actin (sc-1616)

Full unedited gel for Figure 1H

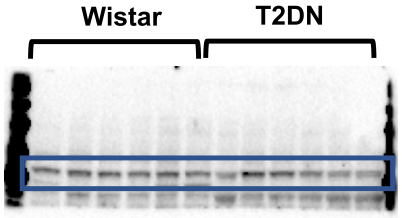

TBK1 (CST38066)

Full unedited gel for Figure 1H

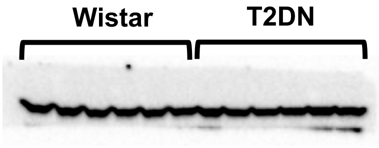

Actin (sc-1616)

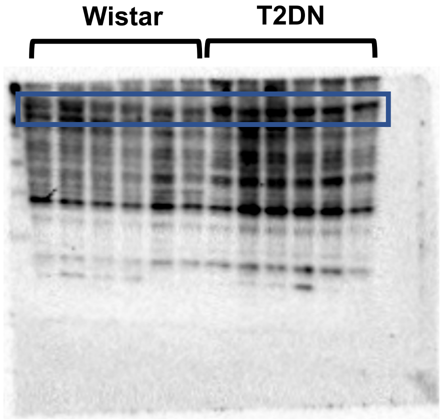

P-IRF3 (CST29047)

Full unedited gel for Figure 1H

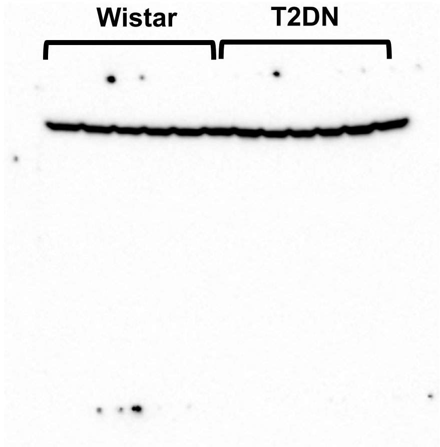

Actin (sc-1616)

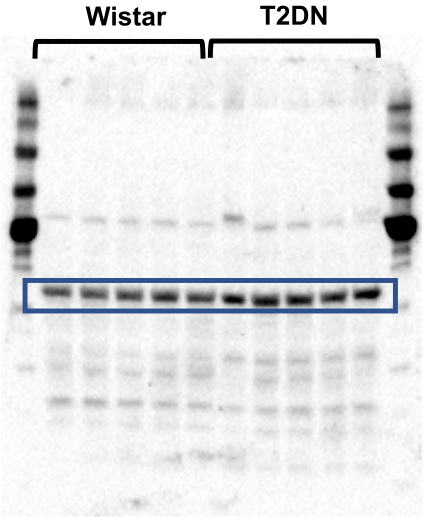

IRF3 (CST4302)

Full unedited gel for Figure 1H

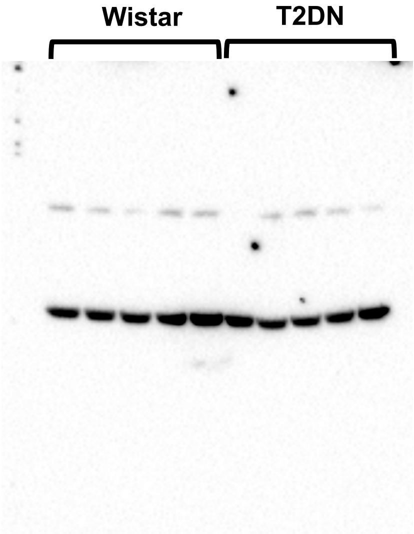

Actin (sc-1616)

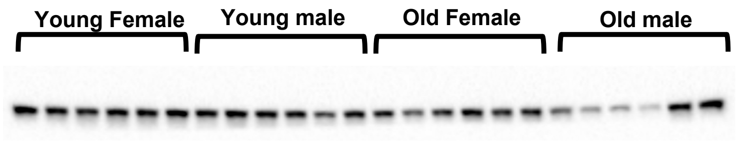

Nephrin (ab5896)

Full unedited gel for Figure 2A

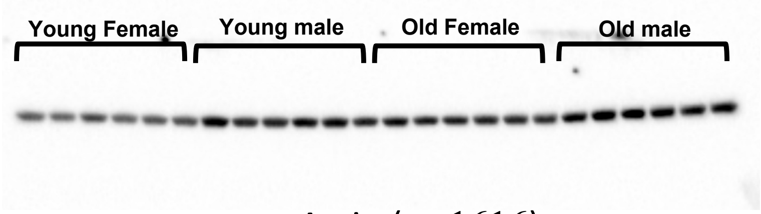

Actin (sc-1616)

Full unedited gel for Figure 2A

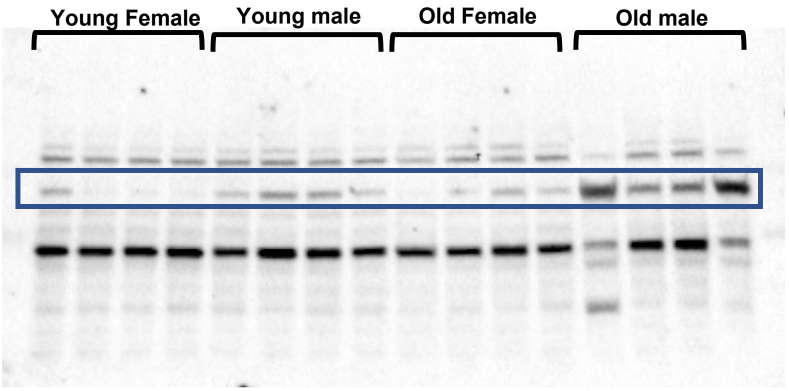

KIM1 (CST14971)

Full unedited gel for Figure 2A

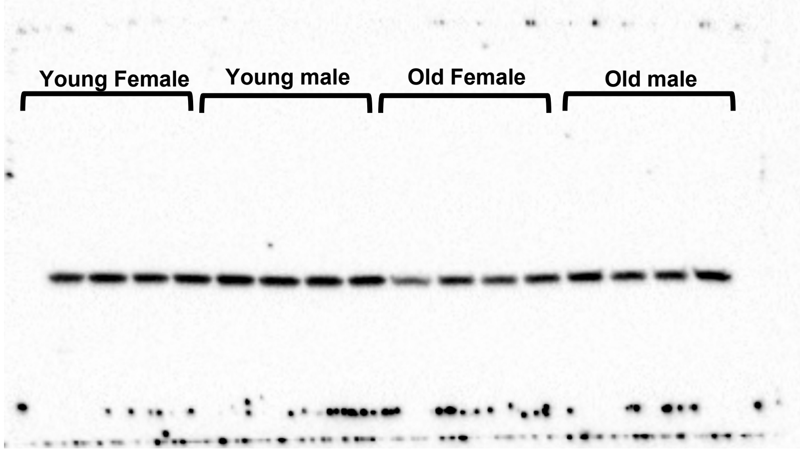

Actin (sc-1616)

Full unedited gel for Figure 2A

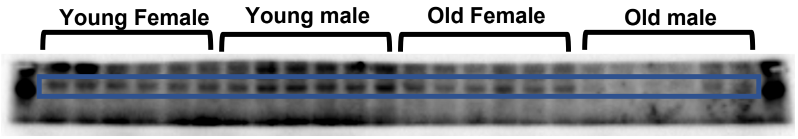

mTFA (ab131607)

Full unedited gel for Figure 3A

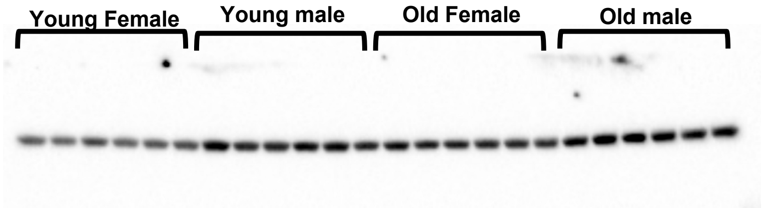

Actin (sc-1616)

Full unedited gel for Figure 3A

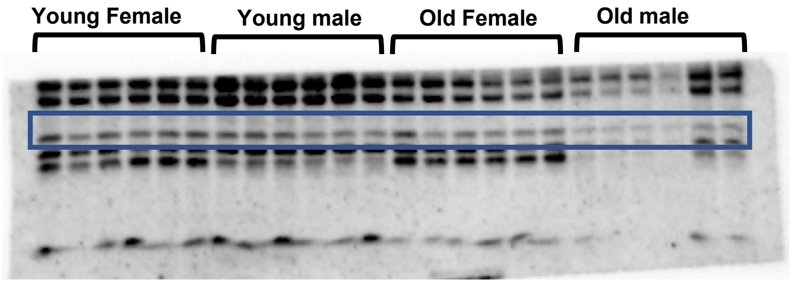

TREX1 (ab83890)

Full unedited gel for Figure 3A

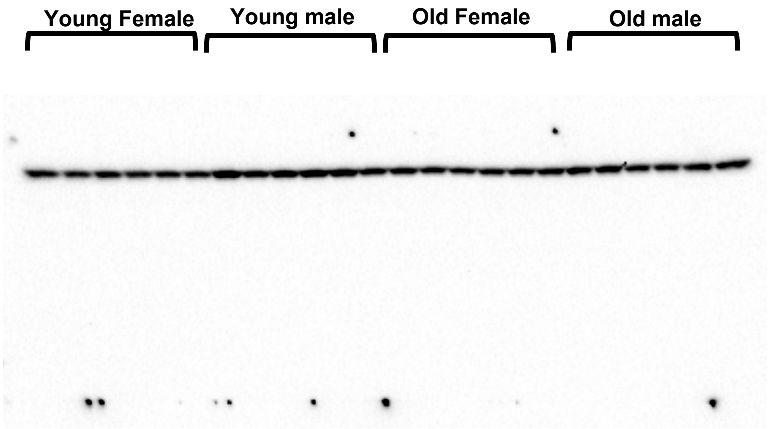

Actin (sc-1616)

Full unedited gel for Figure 3A

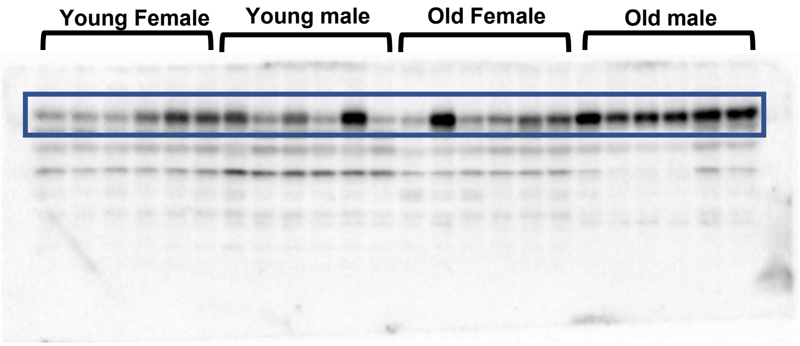

cGAS (MBS8291689)

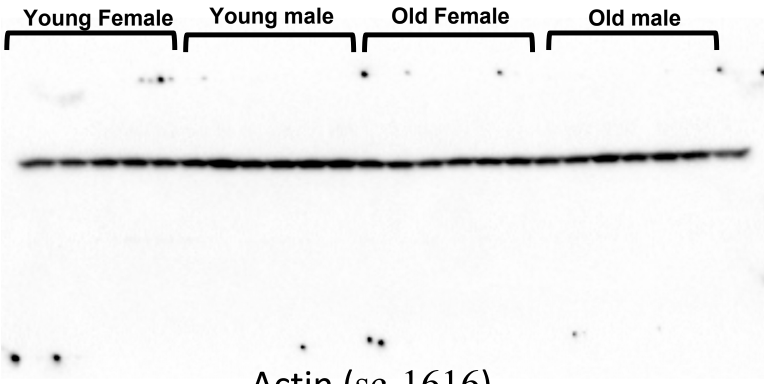

Actin (sc-1616)

Full unedited gel for Figure 3C

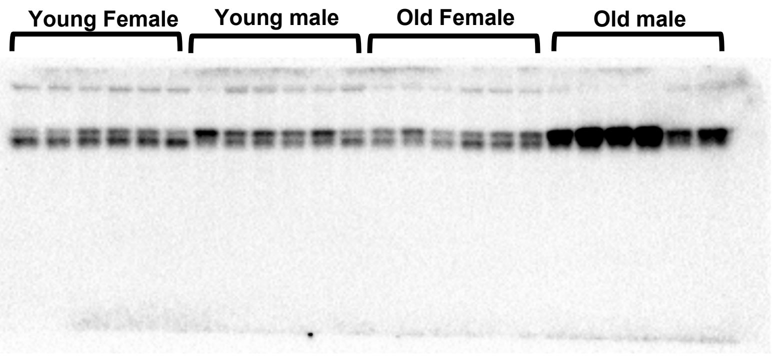

STING (CST50494)

Full unedited gel for Figure 3C

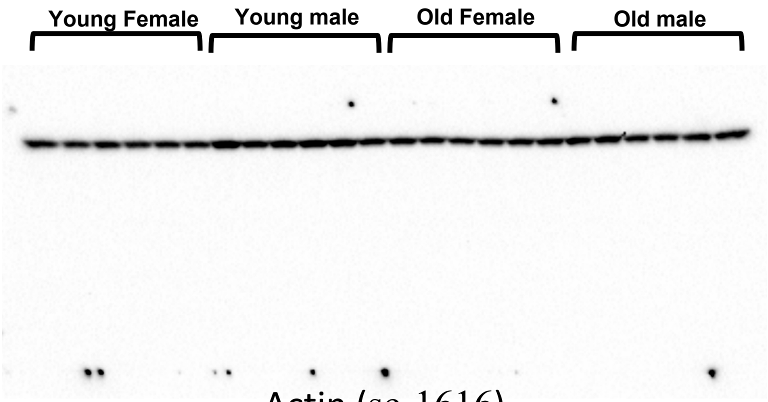

Actin (sc-1616)

Full unedited gel for Figure 3C

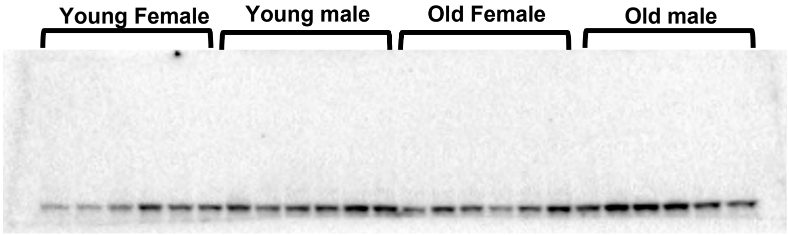

P-TBK1 (CST5483)

Full unedited gel for Figure 3C

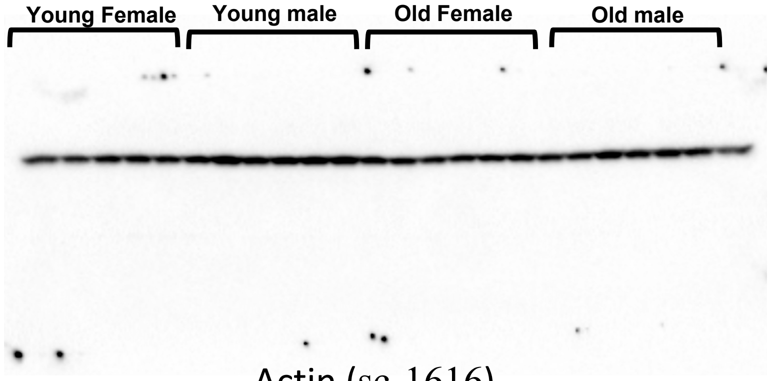

Actin (sc-1616)

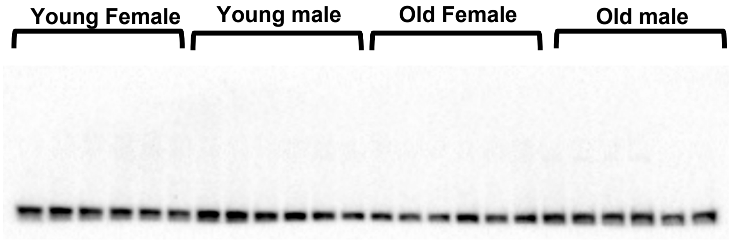

TBK1 (CST38066)

Full unedited gel for Figure 3C

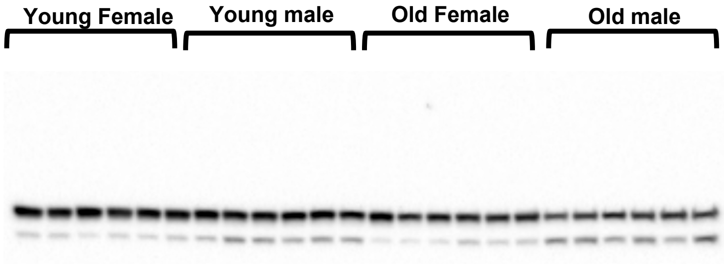

Tubulin (ac030)

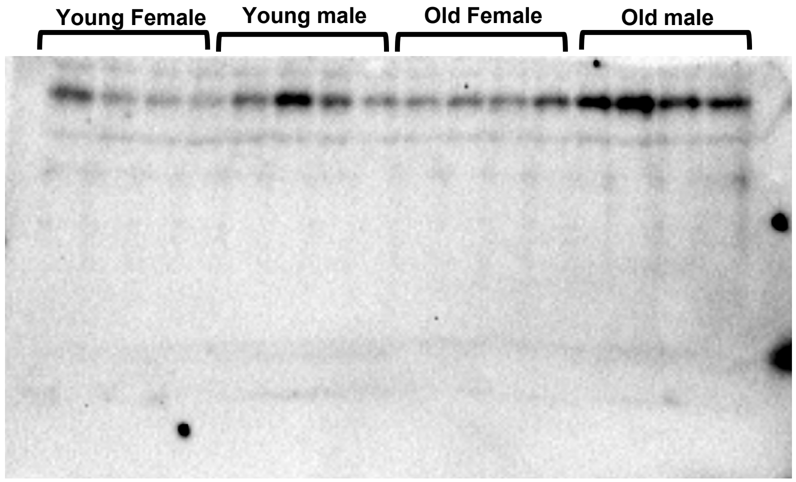

P-IRF3 (CST29047)

Full unedited gel for Figure 3C

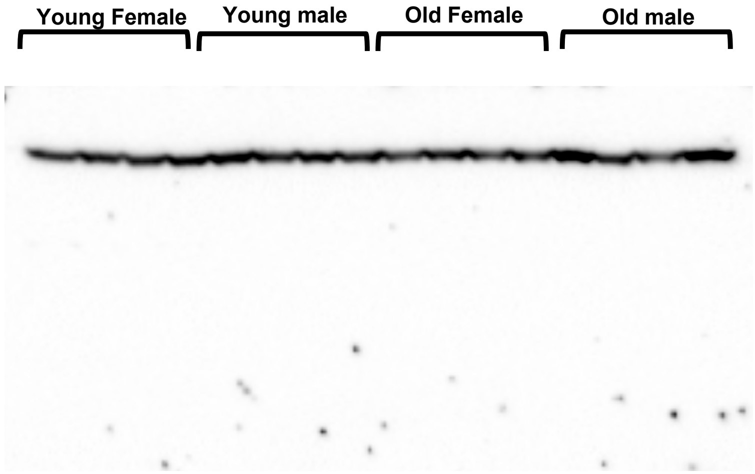

Actin (sc-1616)

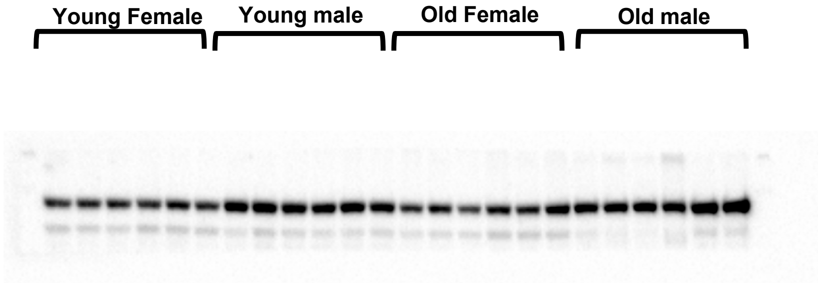

IRF3 (CST4302)

Full unedited gel for Figure 3C

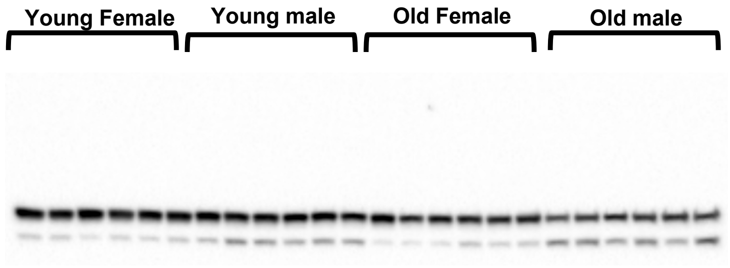

Tubulin (ac030)
